# Effect of 12 Sessions of Short-Duration High-Intensity Interval Training on Executive Function and Inhibitory Control in Healthy Adolescents: A Cluster Randomized Clinical Trial

**DOI:** 10.7759/cureus.101189

**Published:** 2026-01-09

**Authors:** Eduardo Gauze Alexandrino, Vithória Oleiro, Yuri da Gama Rodrigues, Michael Pereira Silva, Fabricio Boscolo Del Vecchio, Samuel Carvalho Dumith

**Affiliations:** 1 Epidemiology and Public Health, Faculty of Medicine, Postgraduate Program in Health Sciences, Federal University of Rio Grande, Rio Grande, BRA; 2 Epidemiology and Public Health, Superior School of Physical Education, Federal University of Pelotas, Pelotas, BRA

**Keywords:** child and adolescent, cognitive performance, executive function, high-intensity interval training (hiit), psychological inhibition, school-based physical activity

## Abstract

There is a gap in the evidence regarding the effects of short-duration high-intensity interval training (short-HIIT) on executive and inhibitory functions in adolescents, particularly when using protocols adapted to the school setting and available infrastructure. Therefore, the aim of this study was to investigate the effects of a short-HIIT program on executive function and inhibitory control in Brazilian school adolescents. This was a randomized, single-center clinical trial with intervention and control groups. Adolescents aged 15-17 years participated in a short-HIIT protocol for six weeks. Primary outcomes were executive function and inhibitory control, assessed using the Trail Making Test (TMT) Parts A and B and the Stroop Color Test, respectively. Data were analyzed per protocol and by intention-to-treat. Intervention effects were examined using generalized estimating equations with linear and Poisson models, incorporating time, group, an interaction term, and adjustment covariates. The significance level for two-tailed tests was set at 5%. The sample included 161 students (46 in the short-HIIT group and 115 in the control group). The main results showed absolute reductions in completion times for both the Stroop and TMTs in both groups, consistent with a potential practice effect, with no statistically significant differences between the intervention and control groups at baseline or post-intervention. After 12 sessions, no statistically significant differences were observed between the intervention and control groups for any of the assessed outcomes, in either crude models or models adjusted for confounding variables (sex, age, asset index, baseline physical activity level, time spent on social media, and BMI). In conclusion, twelve 10-minute sessions of short-HIIT performed at the beginning of physical education classes over six weeks did not alter executive function or inhibitory control in adolescents from southern Brazil. Moreover, the adopted HIIT protocol was safe and was not associated with acute injuries.

## Introduction

Adolescence is a period of intense development in frontoparietal brain regions involved in executive functions, including inhibitory control, cognitive flexibility, and attention [1]. The development of these functions during this stage is critical for academic success and socioemotional regulation, as it enables impulse control, sustained focus, and more deliberate decision-making during a key phase of prefrontal cortex maturation [2]. Physical activity (PA) is widely recognized as a strategy to improve overall adolescent health. However, national and international surveys indicate low levels of PA, which may contribute to various physical and mental health disorders [3-5]. Consequently, the school environment, particularly physical education classes, represents an important strategic setting for large-scale, long-term interventions [6].

Among different exercise modalities, high-intensity interval training (HIIT) has gained attention for delivering health benefits with low time requirements and minimal, low-cost equipment. The short, intense nature of HIIT sessions appears to influence both cognitive capacity and participant adherence [7]. In adolescents, HIIT has been associated with improvements in cardiorespiratory fitness and body composition [8-12], as well as potential neuromuscular benefits [13]. Regarding cognition and mental health outcomes, including depression and anxiety, reviews have identified beneficial effects in youth, although findings are heterogeneous [14-17].

Within the executive domain, the Stroop test (assessing inhibitory control and selective attention) and the Trail Making Test (TMT Parts A and B, assessing processing speed and attentional flexibility) are widely used assessment tools. Recent evidence indicates that HIIT can improve inhibitory control following acute vigorous or intermittent exercise sessions [18-20], whereas acute effects on TMT performance have been less consistent [19]. Interventions lasting four to eight weeks may produce cognitive benefits; however, the magnitude and consistency of these effects depend on exercise dose, study design, and assessment instruments, with frequent heterogeneity observed across intervention protocols [2,21,22]. In school-based HIIT programs, improvements in health-related outcomes have been reported, but evidence for cognitive outcomes remains mixed, highlighting the need for clear, comparable protocols [16,23] and, above all, progressive and reproducible stimuli [24].

In Brazil, studies in adolescents have demonstrated cardiometabolic benefits of HIIT in individuals with obesity [25,26], supporting the feasibility of implementing this model within the national context, including public health and school settings. Despite the premise that HIIT provides comprehensive health benefits [9], cognitive outcomes assessed by the Stroop and TMT remain underexplored in structured, school-based interventions. Improvements in executive function and inhibitory control contribute to better quality of life; nevertheless, a gap exists in the literature regarding the effects of HIIT on these outcomes in adolescents, using protocols feasible for the Brazilian school environment. Therefore, this study aimed to investigate the effects of a HIIT program on executive function and inhibitory control in school adolescents from southern Brazil.

## Materials and methods

Experimental design 

This phase II, single-center, cluster randomized clinical trial was conducted in Rio Grande, Rio Grande do Sul, Brazil, from early June to August 2024. The study was approved by the Research Ethics Committee of the Federal University of Rio Grande (FURG) (approval number 5.846.123; CAAE: 65508322.8.0000.5324). The research adhered to CONSORT (2009) recommendations and was registered in the Brazilian Registry of Clinical Trials (ReBEC: RBR-733y6zq).

The total study duration was 10 weeks, divided into two standardized assessment periods: Weeks 1-2 (baseline, prior to the six-week short-HIIT intervention) and Weeks 9-10 (post-intervention). Outcomes included executive function, cardiorespiratory capacity, and anthropometric measures. Outcome assessment was blinded: evaluators responsible for data collection and those performing data analyses were unaware of group allocation. Blinding of participants and the researchers delivering the intervention was not feasible. The control group did not receive any intervention from the research team and continued scheduled physical education activities, which were identical to those of the intervention group.

Participants, sample, and eligibility criteria 

The study was conducted at the Federal Institute of Education, Science, and Technology of Rio Grande (IFRS) - Campus Rio Grande, Brazil, involving students enrolled in integrated high school programs. Due to logistical and institutional constraints, the sample comprised six second-year classes of both sexes, with a mean age of 16 ± 0.5 years, whose students agreed to participate.

The six classes were randomized in a 1:2 ratio (intervention:control) to maximize statistical power and include all students from the same grade. Student distribution was proportional across classes, and all classes followed the same physical education curriculum. Classes were randomly allocated by a simple draw of sealed envelopes into two groups: the short-HIIT group (n = 2 classes) and the control group (n = 4 classes).

All students regularly attending physical education classes and deemed fit for PA were eligible, except those with medical limitations or previously documented physical disabilities. Students aged 18 years or older or requiring adaptations participated in the intervention but were excluded from statistical analyses.

All participants and their legal guardians were adequately informed about study procedures, and written informed consent was obtained. The total number of students initially selected was 200. Sample size calculation accounted for the cluster design (classes), with a 1:2 allocation ratio, an average class size of 35 students, a two-tailed α of 0.05, 80% power, and an intraclass correlation coefficient (ICC) of 0.02. The ICC was based on prior school-based trials, in which cognitive and psychosocial outcomes typically present low ICCs (~0.01-0.05) [26]. With two classes in the intervention group and four in the control group, the estimated minimum detectable effect size was d = 0.53. Variability in cognitive outcomes was estimated using data from a Brazilian study applying the Stroop test in adolescents with pre- and post-intervention measures following vigorous exercise [27]. Accordingly, the study was powered to detect moderate-to-large effects in the primary outcomes (Stroop interference time and TMT performance).

Study variables 

The independent variable was the short-duration high-intensity interval training (short-HIIT) program. Cognitive outcomes were assessed by a trained team of researchers and psychologists before and after the six-week intervention. Inhibitory control, measured as execution time for each card (seconds), total execution time (seconds), number of errors, and Stroop effect, was evaluated using the Stroop Color Test (Victoria version) [28]. Executive function, measured as execution time for each task and total time, was assessed using the TMT (Parts A and B) [29]. The Brazilian Federal Council of Psychology permits the use of non-standardized tests in the Brazilian population for research purposes (CFP Resolution No. 001/2003, Article 16).

To control for potential confounders, the following variables were included in the analyses: self-reported sex (male/female); age (in complete years); asset index divided into quartiles (constructed from household assets, including air conditioning, car, motorcycle, computer, dryer, freezer, number of bathrooms, and presence of a cleaning professional; each variable had a coefficient >0.25 in the covariance matrix, and the first component was extracted, explaining 33.6% of total variability, Eigenvalue = 2.7); baseline PA level measured using an adapted version of the National School Health Survey (PeNSE) questionnaire [4], with participants considered physically active if reporting ≥300 minutes/week of PA; body weight (kg); self-reported daily time spent on social media, categorized as ≤2 hours/day, 2-4 hours/day, and >4 hours/day; and cardiorespiratory fitness, estimated by VO₂max calculated from maximal aerobic running speed (km/h) obtained from the 20-m shuttle run test and adjusted for age [30].

Short-HIIT protocol

The intervention group participated in 12 sessions of short-HIIT, conducted twice weekly over six weeks. Each session lasted 10 minutes and took place at the beginning of physical education classes in a sports gymnasium (40 × 20 m) with adequate infrastructure for physical exercise.

The protocol followed principles of progressive intensity based on maximal aerobic speed (MAS), previously estimated using the 20-m shuttle run test. Exercise intensity reached at least 80% of maximal heart rate (HRmax), with a 48-hour interval between sessions. Short-HIIT sessions consisted of repeated running bouts in which participants, divided into three groups according to their MAS, were required to reach a marker (cone) at each auditory signal. Heart rate monitors (Polar, model H10^®^, Kempele, Finland) were worn by all participants and monitored in real time to ensure target intensity. At the end of each session, rating of perceived exertion was assessed using the Rating of Perceived Exertion Scale [30]. A detailed description of intensity progression and rest intervals for each session has been reported previously [23].

The control group continued their regular physical education classes, following activities planned by the teachers, without any intervention from the research team. These activities focused on bodyweight exercises and general physical conditioning and were also implemented with the intervention group after the short-HIIT sessions. Participants who did not achieve a minimum attendance of 66% across the 12 sessions were excluded from the analyses.

Statistical analysis

Study data were collected and managed using REDCap^®^ electronic data capture tools hosted at the FURG server by closed-ended questionnaires administered on tablets. REDCap (Research Electronic Data Capture) is a secure, web-based software platform designed to support data capture for research studies [31]. The database, containing self-reported questionnaires, cognitive test results, and cardiometabolic data from each session, was exported to Excel (Microsoft Corporation, Redmond, WA, USA) for organization. Statistical analyses were performed using Stata version 18.0 (StataCorp LLC, College Station, TX, USA).

Continuous variables were presented as means and SDs, and categorical variables as absolute and relative frequencies. Data were analyzed both per protocol and by intention-to-treat. Intervention effects were evaluated using Generalized Estimating Equations, applying linear and Poisson models, incorporating time, group, the interaction term (time × group), and adjustment covariates (sex, age, asset index, baseline PA level, time spent on social media, and BMI).

Mean differences between the intervention and control groups were calculated with corresponding 95% CI, and effect sizes (Cohen’s d) were reported to assess the magnitude and precision of the estimated effects. Statistical significance was set at a two-tailed α level of 0.05.

## Results

**Figure 1:**
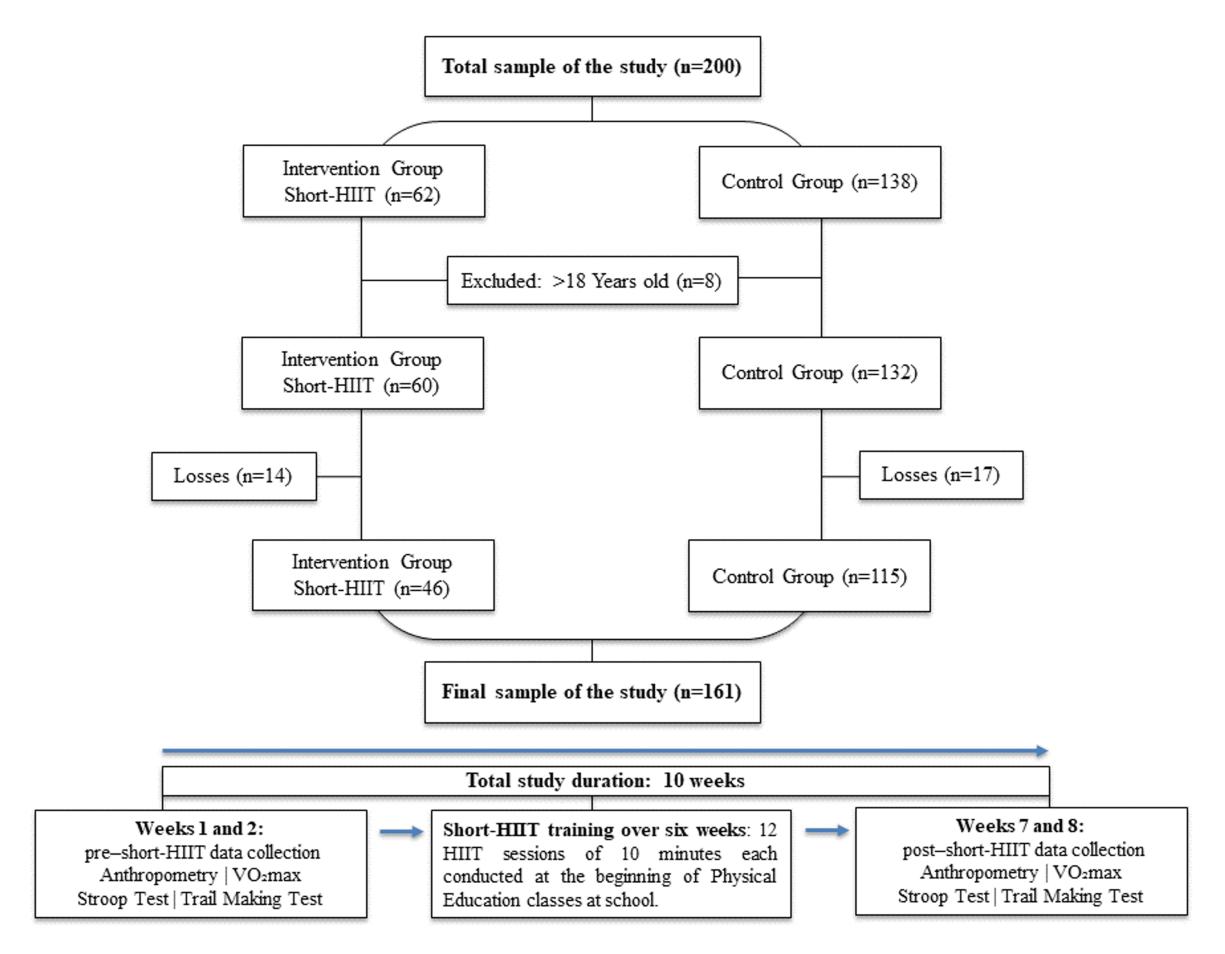
Flowchart of participant enrollment, allocation, and inclusion in analyses HIIT, high-intensity interval training; short-HIIT, short-duration high-intensity interval training; TMT, Trail Making Test

The initial study sample included 200 students, of whom eight were aged 18 years or older and were excluded from the analyses, resulting in a total of 192 adolescents (132 in the control group and 60 in the intervention group). After further exclusions of participants aged 18 years or older and accounting for losses to follow-up (16.1%), the final analytical sample comprised 161 adolescents aged 15-17 years, with 71.4% allocated to the control group (n = 115) and 28.6% to the intervention group (n = 46) (Figure 1).

Table 1 presents the sociodemographic profile of the participants, of whom 52.5% were female, with a mean age of 16.1 ± 0.5 years. Only 13.7% were physically active (≥300 min/week), 28% reported using social media for more than 4 hours per day, and the mean VO₂max was 35.4 ± 4.9 mL·kg⁻¹·min⁻¹. When comparing groups, the intervention group had a higher proportion of males (60.9% vs. 42.1%; p = 0.04) and a slightly higher mean age (16.3 ± 0.4 vs. 16.1 ± 0.5 years; p = 0.02), both statistically significant. No significant differences were observed between groups for the remaining variables, and adherence to the HIIT protocol was 66.5% (≥8 sessions completed).

**Table 1:** Sociodemographic, behavioral, and physical fitness characteristics of school adolescents from southern Brazil at baseline in 2024 (n = 161) ^*^ Fisher’s exact test was used for proportions, and t-test for means. VO₂max is expressed in mL·kg⁻¹·min⁻¹. BMI is expressed in kg/m². PA, physical activity

Variable	All (n = 161)	Control group (n = 115)	Intervention group (n = 46)	p-Value^*^
	N (%)	N (%)	N (%)	
Sex				
Male	76 (47.5)	48 (42.1)	28 (60.9)	0.04
Female	84 (52.5)	66 (57.9)	18 (39.1)	
Age, years (mean ± SD)	16.1 (0.5)	16.1 (0.5)	16.3 (0.4)	0.02
Asset index z-score (mean ± SD)	0.05 (1.7)	0.08 (1.8)	-0.02 (1.5)	0.74
PA				
Inactive (0 min/week)	56 (34.8)	43 (37.4)	13 (28.3)	0.48
Insufficiently active (1-299 min/week)	83 (51.5)	56 (48.7)	27 (58.7)	
Active (≥300 min/week)	22 (13.7)	16 (13.9)	6 (13.0)	
Screen time (social media use)				
Up to 2 hours/day	74 (46.0)	49 (42.6)	25 (54.4)	0.42
2-4 hours/day	42 (26.0)	32 (27.8)	10 (21.7)	
More than 4 hours/day	45 (28.0)	34 (29.6)	11 (23.9)	
VO₂max (mean ± SD)	35.4 (4.9)	35.3 (5.0)	35.6 (4.7)	0.7
BMI (mean ± SD)	23.7 (5.5)	23.5 (5.5)	24.3 (5.2)	0.45

Table 2 presents execution times (mean ± SD) for each phase of the TMT (A/B) and the Stroop Test at baseline and after the short-duration HIIT intervention. Between-group analyses revealed no statistically significant differences in execution times for either the TMT A/B or the Stroop Test, both at baseline and after the intervention. At baseline, both groups showed similar mean values for the TMT (total execution time, p = 0.28), Stroop Effect 1 (p = 0.42), Stroop Effect 2 (p = 0.40), total execution time (p = 0.18), and total errors (p = 0.24). After the intervention, the pattern remained the same, with no differences between groups.

**Table 2:** Comparison of executive function and inhibitory control before and after the intervention in school adolescents from southern Brazil, 2024 (n = 161) ^*^ Independent samples t-test was used for between-group comparisons All times are reported in seconds. TMT (total time): execution time of the three sheets; TMT effect (B-A): execution time of sheet 3 (B) minus the sum of sheets 1 and 2 (A); TMT A: execution time of sheets 1 and 2; Stroop Effect 1: card 3 execution time minus card 1 execution time; Stroop Effect 2: card 3 execution time minus card 2 execution time. short-HIIT, short-duration high-intensity interval training; TMT, Trail Making Test

Cognitive test	Before short-HIIT (mean ± SD)		p-Value^*^	After short-HIIT (mean ± SD)		p-Value^*^
	Control	Intervention		Control	Intervention	
Trails (total time)	121.8 (53.5)	112.1 (44.4)	0.28	91.6 (35.1)	84.5 (29.0)	0.23
Trails effect (B-A)	16.4 (29.4)	18.2 (29.2)	0.72	21.2 (12.1)	19.1 (11.7)	0.3
Trails B (sheet 3)	69.1 (28.5)	65.2 (30.9)	0.45	56.4 (19.9)	53.0 (16.3)	0.32
Trails A (sheet 1 + sheet 2)	52.7 (32.4)	47.0 (21.5)	0.27	35.2 (17.2)	33.8 (12.7)	0.62
Trails (sheet 1)	33.9 (26.8)	28.4 (15.6)	0.19	20.3 (11.0)	19.6 (7.7)	0.69
Trails (sheet 2)	18.8 (8.4)	18.6 (10.1)	0.9	14.9 (7.0)	14.2 (6.1)	0.56
Stroop (total time)	59.2 (13.0)	56.2 (11.2)	0.18	53.0 (10.9)	50.6 (10.7)	0.21
Stroop (total errors)	1.4 (1.7)	1.8 (2.3)	0.24	1.0 (1.3)	1.2 (1.5)	0.5
Stroop effect 1	6.8 (6.2)	5.9 (6.2)	0.42	6.8 (4.8)	6.5 (3.8)	0.69
Stroop effect 2	6.5 (4.4)	5.9 (4.3)	0.4	6.1 (4.0)	5.3 (3.3)	0.24
Stroop time - card 1	17.4 (4.7)	16.8 (5.4)	0.49	15.2 (3.8)	14.3 (3.6)	0.2
Stroop time - card 2	17.6 (4.3)	16.8 (4.4)	0.27	15.9 (3.1)	15.5 (3.3)	0.5
Stroop time - card 3	24.1 (6.3)	22.7 (5.0)	0.16	22.0 (5.6)	20.8 (5.0)	0.22

For the TMT (total time), execution times decreased in both groups (84.5 ± 29.0 vs. 91.6 ± 35.1), but the difference was not statistically significant (p = 0.23). Similarly, no significant differences were observed after short-duration HIIT for Stroop Effect 1 (6.5 ± 3.8 vs. 6.8 ± 4.8; p = 0.69), Stroop Effect 2 (5.3 ± 3.3 vs. 6.1 ± 4.0; p = 0.24), total execution time (50.6 ± 10.7 vs. 53.0 ± 10.9; p = 0.21), or total errors (1.2 ± 1.5 vs. 1.0 ± 1.3; p = 0.50). Even after adjustment, both groups exhibited an absolute reduction in execution times, consistent with a practice effect; however, no statistically significant differences were found between the intervention and control groups at baseline or after the intervention.

Table 3 shows that six weeks of short-HIIT (12 sessions) did not produce statistically significant effects between the intervention and control groups for any of the assessed outcomes (executive function and inhibitory control), in both crude models and models adjusted for potential confounders (sex, age, asset index, baseline PA, time spent on social media, and BMI).

**Table 3:** Effect of six weeks of short-HIIT on executive function and inhibitory control in school adolescents from southern Brazil, 2024 (n = 161) ^*^ Independent samples t-test for between-group comparisons. ^**^ Multiple linear regression adjusted for sex, age, asset index, baseline PA level (inactive, insufficiently active, active), social media use (≤2 h/day, 2-4 h/day, >4 h/day), and BMI. ^***^ Execution time and post-pre short-HIIT time difference, expressed in seconds TMT (total time): execution time of the three sheets; TMT effect (B-A): execution time of sheet 3 (B) minus the sum of sheets 1 and 2 (A); TMT A: execution time of sheets 1 and 2; Stroop Effect 1: card 3 execution time minus card 1 execution time; Stroop Effect 2: card 3 execution time minus card 2 execution time. PA, physical activity; short-HIIT, short-duration high-intensity interval training; TMT, Trail Making Test

Cognitive test	Control (mean ± SD)	Intervention (mean ± SD)	p-Value^*^	Adjusted p-Value^**^
	Difference (post-pre)^***^	Difference (post-pre)^***^		
Trails (total time in seconds)	-30.2 (40.2)	-27.6 (33.8)	0.71	0.94
Trails A (sheet 1 + sheet 2)	-17.5 (25.4)	-13.2 (17.1)	0.29	0.54
Trails B (sheet 3)	-12.7 (22.9)	-11.7 (23.9)	0.81	0.85
Trails B-A	4.9 (27.0)	0.6 (28.7)	0.38	0.62
Stroop (total time in seconds)	-6.2 (9.7)	-5.6 (7.9)	0.74	0.84
Stroop (total errors)	-0.4 (1.9)	-0.6 (2.6)	0.53	0.53
Stroop effect 1	0.0 (6.0)	0.6 (6.5)	0.6	0.35
Stroop effect 2	-0.4 (4.8)	-0.5 (5.5)	0.86	0.88

For the TMT (total execution time), both groups showed similar absolute reductions (intervention: -27.6 ± 33.8; control: -30.2 ± 40.2), with no statistically significant between-group difference (p = 0.71). In the Stroop Test, Stroop Effect 1 showed a mean change of 0.6 ± 6.5 in the intervention group and 0.0 ± 6.0 in the control group (p = 0.60). For Stroop Effect 2, the corresponding changes were -0.5 ± 5.5 and -0.4 ± 4.8, respectively (p = 0.86). Total execution time decreased in both groups (intervention: -5.6 ± 7.9; control: -6.2 ± 9.7), but the difference was not statistically significant (p = 0.74). Regarding total errors, reductions of -0.6 ± 2.6 in the intervention group and -0.4 ± 1.9 in the control group were observed (p = 0.53).

Notably, in the per-protocol analysis, between-group differences increased slightly; however, they remained statistically nonsignificant.

Table 4 shows minimal differences between groups, with small effect sizes (Cohen’s d ranging from 0.03 to 0.19) for executive function and inhibitory control. The lack of statistical significance appears to reflect either the absence of an intervention effect or limitations in the sensitivity of the instruments used to assess cognitive outcomes, rather than insufficient statistical power.

**Table 4:** Magnitude of the effects of short-HIIT on executive function and inhibitory control in school adolescents from southern Brazil, 2024 (n = 161) ^*^ Between-group differences were calculated based on post-pre changes, expressed as mean differences with 95% CIs. ^**^ Effect sizes were estimated using Cohen’s d. short-HIIT, short-duration high-intensity interval training

Cognitive test	Intervention-control difference^*^	Cohen’s d^**^
	Mean (95% CI)	
Trails (total time)	2.6 (-10.7; 15.8)	0.07
Trails A (sheet 1 + sheet 2)	4.4 (-3.7; 14.4)	0.19
Trails B (sheet 3)	1.0 (-7.1; 9.1)	0.04
Trails B-A	-4.3 (-13.9; 5.4)	0.16
Stroop (total time)	0.5 (-2.7; 3.7)	0.06
Stroop (total errors)	-0.2 (-1.0; 0.5)	0.11
Stroop effect 1	0.6 (-1.6; 2.7)	0.09
Stroop effect 2	-0.2 (-1.9; 1.6)	0.03

## Discussion

This randomized clinical trial evaluated the effects of twelve sessions (six weeks) of short-HIIT on executive function and inhibitory control in 161 school adolescents from southern Brazil. The main findings showed that, despite good adherence (75.5%) and methodological rigor, no statistically significant differences were observed between the control group and the short-HIIT group for the assessed cognitive outcomes.

The findings of the present study are consistent with previous investigations that also failed to demonstrate statistically significant effects of several weeks of HIIT on cognitive outcomes in youth. In the analysis of pre- and post-intervention differences (Table 3), reductions of 27.6 seconds in the intervention group and 30.2 seconds in the control group were observed for the TMT (total execution time), with no significant between-group difference (p = 0.71). Similar results were reported by Costigan et al., who evaluated 149 adolescents after eight weeks of school-based HIIT and observed significant improvements in cardiorespiratory fitness, but not in TMT-B performance (mean reduction of 20 seconds) [22]. Duncombe et al. (2022) analyzed 19 trials involving 1,247 participants and reported that HIIT produced robust physical gains but inconsistent effects on cognitive domains related to attention and processing speed [11]. A meta-analysis of 11 studies including 707 adolescent participants found that, compared with other exercise modalities, HIIT promoted greater neuromuscular and anaerobic performance gains but had no impact on simple executive tasks [12]. The absolute reductions in execution times observed for the Stroop and TMTs in the present study, without statistical significance, may reflect a practice or familiarization effect rather than training-induced cognitive improvement, as reported in previous studies [11,12,22].

Conversely, other studies have demonstrated that a single session of intense exercise may influence inhibitory control and cognitive flexibility. Cooper et al. [18] evaluated 56 adolescents exposed to an acute session of high-intensity intermittent games (~85-90% HRmax) and observed acute improvements in Stroop performance immediately after and 45 minutes following the stimulus, particularly among participants with higher cardiorespiratory fitness, indicating a positive acute effect of HIIT on inhibitory control. Hatch et al. [19] assessed 52 adolescents to compare the acute effects of 30 and 60 minutes of HIIT and reported mean Stroop times of 47 ± 9 seconds and 46 ± 8 seconds, respectively, with no differences between groups, values comparable to those observed in the present study (50.6 ± 10.7 seconds in the intervention group and 53.0 ± 10.9 seconds in the control group). The authors concluded that 30 minutes of HIIT was more favorable for adolescent cognition immediately after and 45 minutes post-exercise compared with 60 minutes. A clinical trial [13] involving 305 schoolchildren aged 7-13 years in New Zealand evaluated an eight-week HIIT protocol and observed a mean reduction of seven seconds in Stroop performance, suggesting that longer high-intensity protocols may be required to achieve measurable effects. However, the effect magnitude varied according to fitness level, with less physically fit participants experiencing greater benefits in inhibitory control, in addition to improvements in working memory. In a meta-analysis including 21 studies with more than 1,000 adolescents, small effects on executive functions (d = 0.25) were observed, dependent on longer intervention duration and higher training frequency [6].

Systematic reviews reinforce that exposure time to HIIT is a key determinant of cognitive outcomes [2]. The meta-analysis by Reyes-Amigo et al. [6] reported that most protocols showing significant effects on cognitive variables had durations of at least eight weeks and frequencies of three or more sessions per week. Another meta-analysis of 33 randomized controlled trials in youth identified significant cognitive improvements only in programs lasting longer than eight weeks or with weekly frequencies greater than three sessions for executive function [1]. Other evidence supports that HIIT may generate acute and short-term cognitive benefits; however, these effects tend to be modest and depend on training frequency, duration, and the type of cognitive task employed [1,20]. Regarding the relationship between physical performance and executive function, HIIT has been shown to improve anaerobic performance and motor coordination, but not TMT-A/B execution times, suggesting neuromuscular adaptation specificity rather than cognitive adaptation [12]. Taken together, evidence from previous studies suggests that the total stimulus volume used in the present study (two weekly sessions of 10 minutes, totaling 12 sessions over six weeks) may not have been sufficient to induce cognitive adaptations (executive function and inhibitory control) detectable by psychometric instruments such as the Stroop and TMT.

Despite the absence of statistically significant effects on execution times and cognitive variables, it is important to highlight that implementation of this protocol may confer other health-related benefits in adolescents. Short-HIIT appears to have the potential to promote parallel benefits in physical and mental health, given the integrated nature of exercise-induced adaptations. A clinical trial involving 54 participants reported an 11% increase in VO₂max after 12 weeks of HIIT [7]. A meta-analysis of 23 randomized controlled trials in adolescents reported mean gains of +3.5 mL·kg⁻¹·min⁻¹ in cardiorespiratory fitness [8]. In similar populations, reductions in body fat and improvements in metabolic parameters have also been reported [9,10]. Recent studies have further demonstrated positive effects of HIIT on depressive and anxiety symptoms, with a mean reduction of -3.2 points in depression scores [14]. Ribeiro et al., in an analysis of 1,086 youth, observed a 21% reduction in the relative risk of depression following regular HIIT programs [16]. These findings support the hypothesis that HIIT, even when delivered in short protocols, may generate global health benefits and indirectly influence cognitive and emotional processes. Thus, even in the absence of statistically significant effects on executive function and inhibitory control over six weeks, the present study reinforces the potential of short-HIIT as a strategic, sustainable, low-cost, and high-adherence school-based intervention aimed at promoting overall health and well-being among students [5,23].

Among the strengths of this study are the robustness of the previously published methodological design and the high adherence of participants to the exercise protocol [23]. Second, this study helps address gaps in the scientific literature regarding the effects of six weeks of HIIT in adolescent school populations, as most consistent evidence focuses on acute effects or interventions lasting eight weeks or longer, thereby contributing to future systematic reviews and meta-analyses. Third, the short-duration, low-cost model structured around MAS proved feasible for school implementation without compromising the curriculum, with the objective of promoting overall student health and potential applicability to younger age groups. Fourth, to our knowledge, this is the first Brazilian randomized clinical trial to evaluate the effects of short-HIIT on executive function and inhibitory control in adolescents. Limitations include the single-center design, which limits the generalizability of the findings, and the restricted age range (adolescents only). Additionally, several factors may have limited the emergence and detection of cognitive effects, including the absence of direct neurophysiological measures, the relatively short intervention duration (six weeks, two sessions per week), the brief session length (10 minutes), and the lack of assessment of immediate acute effects of training sessions, which have been evaluated in many acute HIIT studies.

Although statistical significance was not observed, the effects of six weeks of short-HIIT showed small magnitudes (d = 0.03-0.19), suggesting potential clinical relevance. In exercise-based randomized controlled trials, particularly those involving short interventions, the absence of statistical significance does not necessarily imply the absence of a measurable physiological or cognitive effect but may instead reflect the need for a greater exposure dose (total protocol duration) or more sensitive measurement instruments. Thus, the adopted short-HIIT protocol appears promising for promoting parallel improvements in cardiorespiratory fitness, anthropometry, and other cognitive-related variables, although its cognitive benefits may require longer duration, sessions exceeding 10 minutes, and/or higher weekly frequency to become detectable, as indicated by recent meta-analyses.

## Conclusions

Twelve 10-minute sessions of short-HIIT, performed at the beginning of physical education classes over six weeks, did not produce statistically significant effects on executive function (TMT A/B) or inhibitory control (Stroop test) in high school students. However, both groups showed an overall reduction in test execution times, possibly reflecting a practice or familiarization effect with the assessments.

Therefore, future studies are encouraged to adopt the short-HIIT protocol used in the present study, characterized by 10-minute sessions with progressive weekly increases in intensity conducted at the beginning of physical education classes. This intervention structure proved to be feasible, well accepted by students, and easily reproducible in school settings, allowing the evaluation of different intervention durations (eight to 12 weeks) and weekly frequencies (two to three sessions). In addition, the adopted HIIT protocol was safe and was not associated with acute injuries. Finally, the application of this standardized model may help address gaps in the literature by enabling comparisons across studies, facilitating the investigation of acute and chronic cognitive effects, and strengthening the evidence base for incorporating HIIT programs as a school-based strategy for short- and long-term health promotion.
